# VectorNet: Putting Vectors on the Map

**DOI:** 10.3389/fpubh.2022.809763

**Published:** 2022-04-04

**Authors:** Marieta Braks, Francis Schaffner, Jolyon M. Medlock, Eduardo Berriatua, Thomas Balenghien, Andrei Daniel Mihalca, Guy Hendrickx, Cedric Marsboom, Wim Van Bortel, Renate C. Smallegange, Hein Sprong, Céline M. Gossner, Ewelina Czwienczek, Sofie Dhollander, Olivier Briët, William Wint

**Affiliations:** ^1^National Institute of Public Health and the Environment, Utrecht, Netherlands; ^2^Francis Schaffner Consultancy/Mabritec AG, Riehen, Switzerland; ^3^UK Health Security Agency, Porton Down, United Kingdom; ^4^Department of Animal Health, University of Murcia, Murcia, Spain; ^5^UMR ASTRE, CIRAD, University of Montpellier, Montpellier, France; ^6^University of Agricultural Sciences and Veterinary Medicine of Cluj-Napoca, Cluj-Napoca, Romania; ^7^Avia-GIS, Zoersel, Belgium; ^8^Institute of Tropical Medicine, Antwerp, Belgium; ^9^Wageningen Academic Publishers, Wageningen, Netherlands; ^10^European Centre for Disease Prevention and Control, Solna, Sweden; ^11^European Food Safety Authority, Parma, Italy; ^12^Environmental Research Group Oxford Ltd, c/o Dept Zoology, Oxford, United Kingdom

**Keywords:** mosquitoes, ticks, sand flies, biting midges, vector-borne diseases, mapping, geographical distribution

## Abstract

Public and animal health authorities face many challenges in surveillance and control of vector-borne diseases. Those challenges are principally due to the multitude of interactions between vertebrate hosts, pathogens, and vectors in continuously changing environments. VectorNet, a joint project of the European Food Safety Authority (EFSA) and the European Centre for Disease Prevention and Control (ECDC) facilitates risk assessments of VBD threats through the collection, mapping and sharing of distribution data for ticks, mosquitoes, sand flies, and biting midges that are vectors of pathogens of importance to animal and/or human health in Europe. We describe the development and maintenance of this One Health network that celebrated its 10th anniversary in 2020 and the value of its most tangible outputs, the vector distribution maps, that are freely available online and its raw data on request. VectorNet encourages usage of these maps by health professionals and participation, sharing and usage of the raw data by the network and other experts in the science community. For the latter, a more complete technical description of the mapping procedure will be submitted elsewhere.

## Background

Global and local changes for example in land use, social activities, climate, and trade flows, can lead to introductions of vectors and pathogens into new areas, enormous fluctuations, and variations in risk within affected areas, or re-introduction into previously affected areas. When only human or animal disease cases are monitored, information essential to assessing and controlling the vector-borne disease (VBD) risk is missed, for example, pathogens may be present in vectors, but not (yet) in domestic animals or humans. Vectors need to be present before vector-borne transmission of a pathogen can occur, so vector distributions provide the first indications of a VBD risk. Moreover, practical prevention and control of many VBDs relies on reducing the number of (infected) vectors through vector control or exposure mitigation (e.g., through public awareness, limitations in animal movements/exposure). Understanding the presence, absence, abundance, and seasonality of vectors and the pathogens that they transmit is vital to the risk assessment process, which permits effective VBD early warning and control for public as well as veterinary health or jointly in a One Health approach (1, 2).

The value of spatial information on vectors was recognized by the European Center for Disease Prevention and Control (ECDC), who funded the TigerMaps project that produced distribution map projections of the invasion of the Asian tiger mosquito *Aedes albopictus* in Europe (2008–2009) (3). Subsequently, the European Network for Arthropod Vector Surveillance for Human Public Health (Vbornet, 2010–2013) was established to assist ECDC in its VBD preparedness activities by producing pan-European distribution maps of species that vector pathogens. At the same time, various data collection projects were carried out on vector distribution by the European Food Safety Authority (EFSA) to support their risk assessments on VBDs of veterinary importance. ECDC and EFSA subsequently joined forces in the VectorNet project (2014–2018), extending the established network greatly to include entomologists, public health professionals and veterinarians working in the field of VBDs in Europe and neighboring countries. This network for obtaining and sharing data on the geographic distribution of arthropod vectors (mosquitoes, ticks, sand flies, and biting midges) transmitting human and animal disease agents also supported targeted entomological field collections in specific vector habitats to fill knowledge gaps that had been previously identified. These activities have been consolidated in the current iteration of VectorNet (2019–2023).

## Vectornet Entomological Network

VectorNet is now supplemented with a structured entomological network that is harmonized with other networks within the European public and animal health agencies (4). This VectorNet Entomological Network (VEN) encompasses 51 countries: EU/EEA (26+3), EU Enlargement policy (5) and European Neighborhood Policy partner (15) countries. A strong professional VEN is intended to encourage national governments to ensure sustainable vector surveillance.

## VectorNet Maps

### Data Sources

Producing vector distribution maps is complex and time consuming. The data can come from entomologists doing surveys in the field, or from the (un)published accounts of past surveys. Some governments have monitoring programmes to check for vectors at seaports and airports. In most countries, vector surveillance is not mandatory, with no formal reporting system or collection standards. VectorNet supports the establishment and standardization of protocols (e.g.. sampling protocol) and to collect the best available information, through voluntary data sharing by researchers and public health bodies, and through regular literature review.

### VectorNet Data

Vector distribution mapping requires information about the vectors and their location, in the form of the sample coordinates, either as georeferenced point data or linked to territorial units [e.g., Nomenclature of Territorial Units for Statistics (NUTS)]. The former has the advantage of being independent of administrative boundary changes and allows more precise analysis, but it creates more barriers to sharing the data. VectorNet stresses the need for data from surveys that do not find vectors despite appropriate surveillance effort (absence data).

**Figure 1:**
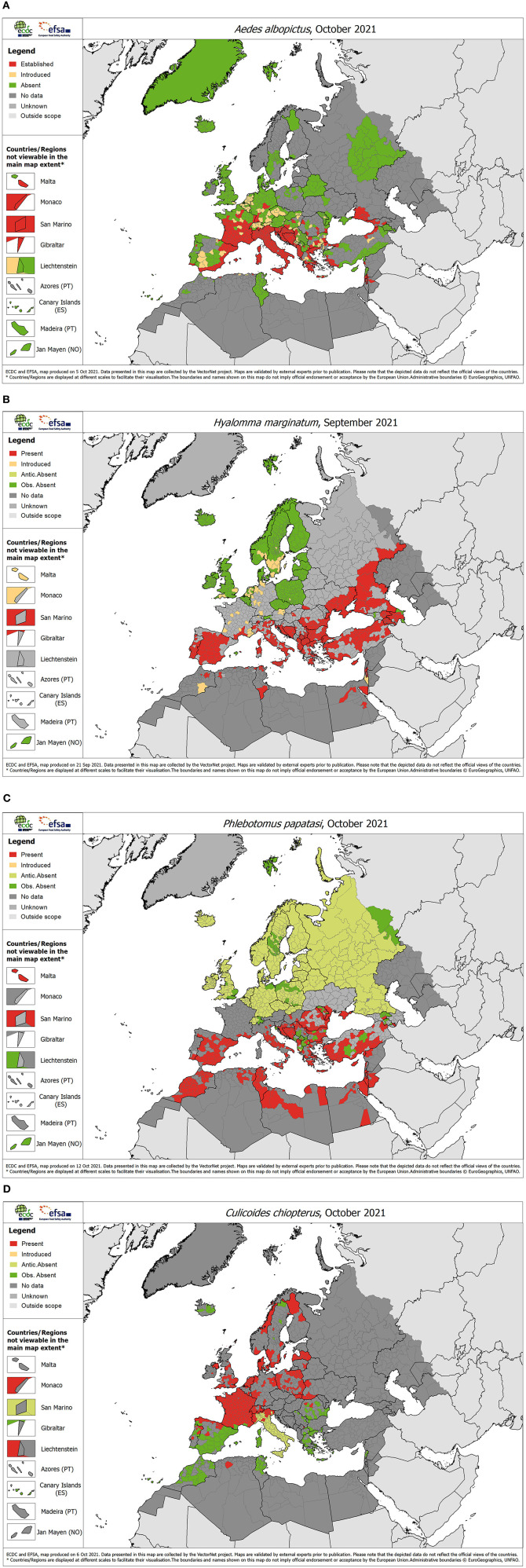
Representative examples of vector distribution maps for each of the four vector groups, October 2021. **(A)** Mosquitoes: *Aedes albopictus*, **(B)** Ticks: *Hyalomma marginatum*, **(C)** Sand flies: *Phlebotomus papatasi*, **(D)** Biting midges: *Culicoides chiopterus*.

From the hundreds of arthropod species possibly involved in pathogen transmission in Europe, a number of species have been prioritized on the basis of their public and veterinary health importance within each of the target groups (7 ticks, 10 midges, 12 sand flies, 9 invasive and 20 native mosquitoes) so that in all, the project compiles information for 58 vector species (Figure 1).

While producing the actual distribution maps is relatively easy, collecting the data into a structured and harmonized database and curating the records is more time-consuming, especially when original data are recorded in different formats. Further, the database requires continuous updates as the distribution status of a species in an administration unit can alter between absent, introduced and established over time due to ecological change or trade, a phenomenon well-known for *Ae. albopictus*.

Over the years, over 150,000 vector location records have been acquired and curated, and regular updates continue to add 20,000 records each year. All records are collated and validated by VectorNet core team members and are subjected to a three-stage error checking process before being added to the archive. VectorNet distribution are stored by ECDC and may be requested *via* https://www.ecdc.europa.eu/en/about-us/document-request or https://connect.efsa.europa.eu/RM/s/askefsa.VectorNet encourages participation, sharing and usage of the raw vector data by VEN, entomologists and other experts in the science community by providing support through accessible bilateral contact and technical reports, including a full technical description of the mapping process used by VectorNet (in development).

The simple presence and absence measures for administrative units, which were the original focus of the VectorNet, have been replaced by an emphasis on recording measures of the number, abundance and sampling effort of vectors at a point location. These more detailed records are more likely to be related to the pathogen transmission dynamics at a certain time and location (6). Collecting such comprehensive abundance data from the field is, however, time and resource demanding, hence expensive because abundance sampling requires much more standardization and repeated sampling than sampling to detect vector presence alone. VectorNet therefore continues to collect presence/absence vector data whilst further developing strategies to unlock and interpret data on abundance and seasonality. In the current project, assembling abundance and seasonality data in the data warehouse are therefore embedded priorities for all species. Besides producing standardized maps and making raw data available, VectorNet also supports the needs of the agencies with targeted interpretation and analysis, exemplified with the series of spatial distribution models for potential mosquito vectors of Rift Valley fever virus (5, 7).

## Value of Vectornet Maps

The easily accessible transboundary distribution information provided by VectorNet enables health professionals to coordinate more effectively with their neighbors, and to understand the risk from beyond their own borders.

The maps give an overview of the current knowledge of where the vectors are considered to be present and therefore where there is a potential for vector-borne pathogen transmission, and where they are not. The continual updating of the maps is important as distributions of arthropod vectors are affected by environmental changes and trade and travel.

The maps are therefore continually evolving as experts share their data. One of the valuable functions of these maps is to show where there are knowledge gaps and to inform stakeholders where field sampling should be implemented.

The maps provide geographical information on presence, absence, or unknown status, summarized for relatively large subnational units so that the maps can be easily visualized. The underlying database contains more detailed information that can be made available on request, which can be used for more complex mapping of, for example, vector abundance, or technical analysis such as spatial modeling.

The VectorNet maps are validated by VEN members and local entomologists, which helps to assure quality and reliability. The multinational nature of the datasets also helps to engender a sense of joint ownership and promote the synergistic value of data sharing between countries and regions. In a sense, this is perhaps the most important impact of this initiative in that it helps to produce outputs that are supported by the professional community at a continental level.

As a joint ECDC/EFSA project, focusing on vectors of public and veterinary health relevance, VectorNet takes a “One Health” approach, facilitating data sharing between sectors. These maps are widely used in presentations by academic and public health professionals and in advocacy for VBD related activities and are trusted by those involved in planning and strategic development of disease preparedness throughout Europe and its neighbors.

## Vectornet Beyond Mapping

Besides the collection of spatial and temporal data on vector distributions, VectorNet provides scientific support on entomological topics to EFSA and ECDC for their joint as well as separate activities related to VBDs, in the form of technical reports (systematic), literature reviews, fact sheets, modeling and advice on risk assessments (8–13). VectorNet also builds One Health capacity in public health experts, animal health experts and entomologists responsible for national surveillance and control through scientific and technical webinars and face-to-face training sessions (available on ECDC Virtual Academy, https://eva.ecdc.europa.eu).

## Conclusion

The vector distribution maps produced by VectorNet and its preceding projects, over the course of more than 10 years, are a valuable “public good” which have become a “go-to” resource for academics, public as well as veterinary health professionals and the media. The project demonstrates the value of data sharing across national borders and between disciplinary silos (One Health) and confirmed the feasibility of collecting complex information at a continental level by enrolling largely voluntary contributions from a large network supported by a small core of project members. In the context of ever-looming environmental and climatic changes, and the globalization of trade and travel, the risks posed by the emergence and spread of VBDs continue to change. VectorNet's maps will continue to be useful for the foreseeable future.

## Data Availability Statement

The maps presented in the study are available on the website https://www.ecdc.europa.eu/en/disease-vectors/surveillance-and-disease-data. Data inquiries can be directed to access.to.documents@ecdc.europa.eu.

## Author Contributions

MB as the coordinator of the VectorNet consortium, and WW as leader of work package responsible of the mapping of vector data within VectorNet Project, drafted the initial manuscript, after discussing perspectives with the other work package leaders WVB, AM, and HS and the project managers OB and CG of ECDC. EC and SD of EFSA, the consortium members FS, JM, EB, and TB that lead the vector groups (mosquitoes, ticks, sand flies and biting midges, respectively). The members of the project's quality assurance team, GH, CM, and RS were consulted on the data processing. All authors reviewed drafts and approved the final version for submission

## Funding

Within the VectorNet project, the work is performed under specific contracts of EFSA (EFSA SC1) and ECDC (SC1 ECD.9918 and SC2 ECD.10468) implementing framework contract No ECDC/2019/020.

## Conflict of Interest

GH and CM are employed by AviaGis and FS by Francis Schaffner Consultancy/Mabritec AG, Riehen. RS is employed by Wageningen Academic Publishers. WW was employed by Environmental Research Group Oxford Ltd. The remaining authors declare that the research was conducted in the absence of any commercial or financial relationships that could be construed as a potential conflict of interest.

## Publisher's Note

All claims expressed in this article are solely those of the authors and do not necessarily represent those of their affiliated organizations, or those of the publisher, the editors and the reviewers. Any product that may be evaluated in this article, or claim that may be made by its manufacturer, is not guaranteed or endorsed by the publisher.
